# Elevated Expression of Serotonin 5-HT_2A_ Receptors in the Rat Ventral Tegmental Area Enhances Vulnerability to the Behavioral Effects of Cocaine

**DOI:** 10.3389/fpsyt.2013.00002

**Published:** 2013-02-06

**Authors:** David V. Herin, Marcy J. Bubar, Patricia K. Seitz, Mary L. Thomas, Gilbert R. Hillman, Yevgeniya I. Tarasenko, Ping Wu, Kathryn A. Cunningham

**Affiliations:** ^1^Center for Addiction Research, University of Texas Medical BranchGalveston, TX, USA; ^2^Department of Pharmacology and Toxicology, University of Texas Medical BranchGalveston, TX, USA; ^3^Department of Neuroscience and Cell Biology, University of Texas Medical BranchGalveston, TX, USA

**Keywords:** 5-HT_2A_ receptor, cocaine, serotonin, virally mediated gene transfer, AAV, ventral tegmental area, locomotor activity, psychostimulants

## Abstract

The dopamine mesocorticoaccumbens pathway which originates in the ventral tegmental area (VTA) and projects to the nucleus accumbens and prefrontal cortex is a circuit important in mediating the actions of psychostimulants. The function of this circuit is modulated by the actions of serotonin (5-HT) at 5-HT_2A_ receptors (5-HT_2A_R) localized to the VTA. In the present study, we tested the hypothesis that virally mediated overexpression of 5-HT_2A_R in the VTA would increase cocaine-evoked locomotor activity in the absence of alterations in basal locomotor activity. A plasmid containing the gene for the 5-HT_2A_R linked to a synthetic marker peptide (Flag) was created and the construct was packaged in an adeno-associated virus vector (rAAV-5-HT_2A_R-Flag). This viral vector (2 μl; 10^9–10^ transducing units/ml) was unilaterally infused into the VTA of male rats, while control animals received an intra-VTA infusion of Ringer’s solution. Virus-pretreated rats exhibited normal spontaneous locomotor activity measured in a modified open-field apparatus at 7, 14, and 21 days following infusion. After an injection of cocaine (15 mg/kg, ip), both horizontal hyperactivity and rearing were significantly enhanced in virus-treated rats (*p* < 0.05). Immunohistochemical analysis confirmed expression of Flag and overexpression of the 5-HT_2A_R protein. These data indicate that the vulnerability of adult male rats to hyperactivity induced by cocaine is enhanced following increased levels of expression of the 5-HT_2A_R in the VTA and suggest that the 5-HT_2A_R receptor in the VTA plays a role in regulation of responsiveness to cocaine.

## Introduction

Cocaine addiction is marked by significant morbidity and loss of human potential, yet consistently effective and accessible recovery options remain limited. This fact underscores the continuing need to uncover the neural factors that drive vulnerability to cocaine addiction and relapse and to establish new pharmacological strategies to halt or reverse the progression of the disorder. Cocaine inhibits reuptake of monoamines, including dopamine (DA) and serotonin (5-hydroxytryptamine; 5-HT; Koe, 1976) and the enhanced efflux of DA within the mesocorticoaccumbens circuit is critical in the generation of cocaine-evoked behaviors (Kelly and Iversen, 1976; Delfs et al., 1990; Callahan et al., 1994). The mesocorticoaccumbens DA neurons, which originate in the ventral tegmental area (VTA) and project prominently to subcortical [e.g., nucleus accumbens (NAc)] and cortical structures [e.g., prefrontal cortex (PFC)], are under the modulatory control of the 5-HT system (Alex and Pehek, 2007), with 5-HT neurons in the dorsal raphe nucleus innervating both cell body and terminal regions of the mesocorticoaccumbens circuit (Halliday and Tork, 1989). As such, the 5-HT system is also an important mediator of cocaine-evoked behaviors (for reviews, see Walsh and Cunningham, 1997; Muller and Huston, 2006; Bubar and Cunningham, 2008; Filip et al., 2010).

The 5-HT_2A_ receptor (5-HT_2A_R), one of 14 subtypes of 5-HT receptors (Hoyer et al., 2002), plays an integral role in mediating the serotonergic influence upon cocaine-evoked behaviors (Muller and Huston, 2006; Bubar and Cunningham, 2008). Blockade of the 5-HT_2A_R with a selective antagonist (e.g., M100907; SR46349B) significantly reduces cocaine-evoked hypermotility (O’Neill et al., 1999; McMahon and Cunningham, 2001; Fletcher et al., 2002; Filip et al., 2004; Szucs et al., 2005), behavioral sensitization (Filip et al., 2004; Zayara et al., 2011), and behavioral disinhibition (Anastasio et al., 2011; Fletcher et al., 2011; Cunningham et al., 2013), as well as the discriminative stimulus effects of cocaine (Filip et al., 2006) and cocaine- (Fletcher et al., 2002) and cue-primed (Nic Dhonnchadha et al., 2009; Pockros et al., 2011) reinstatement of cocaine-seeking in rats. Systemic administration of the non-selective 5-HT_2A_R antagonist ketanserin inhibited cocaine-evoked hypermotility simultaneous with inhibition of cocaine-evoked DA release in the NAc (Broderick et al., 2004), suggesting an important role for 5-HT_2A_R-modulation of DA mesocorticoaccumbens neurotransmission in cocaine-evoked behaviors mediated by this circuit.

The 5-HT_2A_R is a G protein-coupled receptor (Berg et al., 1994) expressed throughout the nodes of the mesocorticoaccumbens circuit (Cornea-Hebert et al., 1999; Doherty and Pickel, 2000; Xu and Pandey, 2000; Nocjar et al., 2002; Miner et al., 2003). The 5-HT_2A_R resident in the VTA is localized to both DA and non-DAergic [presumably γ-aminobutyric (GABA) or glutamate] neurons within the VTA (Doherty and Pickel, 2000; Nocjar et al., 2002), and appear to be integral in modulating psychostimulant-induced behaviors mediated by the mesocorticoaccumbens circuit. Microinfusion of the selective 5-HT_2A_R antagonist M100907 into the VTA, but not the NAc, attenuated hyperactivity evoked by systemic administration of cocaine at doses that did not alter basal motor activation (McMahon et al., 2001). Likewise, intra-VTA 5-HT_2A_R antagonist administration significantly blocked amphetamine-evoked hyperactivity and associated DA release in the NAc, with no effect upon basal motor activity or DA efflux in NAc (Auclair et al., 2004). We have observed that microinfusion of the preferential 5-HT_2A_R agonist 1-(2,5-dimethoxy-4-iodo)-2-aminopropane (DOI) alone into the VTA is sufficient to evoke hyperactivity in rats (Herin et al., unpublished observations). Thus, activation of 5-HT_2A_R resident in the VTA results in behaviorally significant outcomes, and likewise appears to play a critical role in cocaine-evoked behaviors mediated by the DA mesocorticoaccumbens circuit.

The virally mediated gene transfer technique represents a targeted means to manipulate the expression of important proteins in the brains of adult animals (Carlezon et al., 1997; Bolanos et al., 2003; Edry et al., 2011). A recombinant adeno-associated virus (rAAV) can be used to selectively transduce neurons for a long duration (weeks to months) with a minimum of toxicity and inflammation (McCown et al., 1996; Lo et al., 1999). In the present study, we have exploited rAAV-mediated gene transfer to investigate whether overexpression of the 5-HT_2A_R in the VTA alters the vulnerability of adult male rats to the hypermotive effects of cocaine. We developed an rAAV containing the coding region for the 5-HT_2A_R linked to a synthetic marker peptide (Flag; rAAV-5-HT_2A_R-Flag), and infused vehicle or rAAV-5-HT_2A_R-Flag unilaterally into the VTA of experimental animals, followed by measurement of basal and cocaine-evoked hyperactivity. Immunohistochemical analyses were used to confirm 5-HT_2A_R overexpression as well as expression of the Flag peptide.

## Materials and Methods

### Animals

Male Sprague-Dawley rats (Harlan Sprague-Dawley, Inc., Indianapolis, IN, USA) weighed 250–275 g at the beginning of the study. The rats were housed (initially four/cage) in standard plastic rodent cages in a temperature (21–23°C) and humidity (55–65%) controlled environment under a 12-h light/dark cycle (lights on 07:00 h). Animals were acclimated to the colony for 3–5 days prior to surgery, after which they were single-housed and allowed to recover for at least one week prior to the start of experimental sessions. All animals were provided with food and water *ad libitum*. Experiments were conducted during the light phase of the light-dark cycle (1200–1800 h) and were in accordance with the National Institutes of Health *Guide for the Care and Use of Laboratory Animals* and with approval from the UTMB Institutional Animal Care and Use Committee.

### Viral vectors

The cDNA containing the coding region for the rat 5-HT_2A_R was obtained (Dr. J. Liu, University of Cincinnati). Primers were designed to amplify only the coding region of the 5-HT_2A_R and to add a *Bam*HI site (to the 5′ end), *Spe*I site (to the 3′ end), 24 bases coding for a synthetic marker (Flag) protein and a stop codon. PCR amplification was performed using an rTtH-XL polymerase (Perkin Elmer, Boston, MA, USA) and the product (5-HT_2A_R-Flag) was purified by preparative agarose gel electrophoresis followed by dialysis, phenol/chloroform/isoamyl alcohol (25:24:1) extraction and ethanol precipitation.

The 5-HT_2A_R-Flag DNA was then ligated into a pCW plasmid, which is appropriate for viral packaging. The pCW plasmid (provided by Dr. D. J. Poulsen; University of Montana) contains the inverted terminal repeats (ITR) of AAV, a chick β-actin (CAG) promoter, multiple cloning sites, and a woodchuck hepatitis virus posttranscriptional regulatory element (WPRE; Stone et al., 2005). 5-HT_2A_R-Flag (300 fmol) and pCW (30 fmol) were digested with *Bam*HI (2 units) and *Spe*I (5 units) for 2 h at 37°C. Ligation was performed with a kit (TaKaRa Biochemical, Inc., Berkeley, CA, USA). Ten microliters of the resultant cDNA was used to transform DH5-α ultracompetent *E. coli* containing 50 μg/ml ampicillin. Plasmid DNA was isolated from 20 colonies and tested for inclusion of the plasmid by digestion with *Bam*HI and *Spe*I. One positive colony was CsCl-purified and sequenced at the UTMB Molecular Biology Core Facility. Functionality of the transgene was determined by transfection in raphe RN46A cells (provided by Dr. Scott Whittemore, University of Miami; White et al., 1994) followed by immunocytochemical detection with 5-HT_2A_R and Flag antibodies.

The rAAV-5-HT_2A_R-Flag was prepared by cotransfecting three plasmids into human embryonic kidney cells (HEK 293 cell line) based on previous protocols (Xiao et al., 1998; Wu et al., 2002) utilizing an AAV helper plasmid (pXX2) and an adenovirus helper plasmid (pXX6). The HEK cells were cultured in 150 mm dishes containing DMEM/10% FBS at 37°C, 5% CO_2_. When cells reached 80% confluence, calcium phosphate precipitation was used for co-transfection with pCW-5-HT_2A_R-Flag, pXX2, and pXX6. Following a brief rinse with DMEM, OptiMEM (Life Technologies)/10% FBS/120 μM chloroquine was added to the cells. Then 2.5 ml of DNA–calcium phosphate solution was added per plate. This solution contained three plasmids at the molar ratio of 7:2:4, 125 mM CaCl_2_ and 1× HBS (2.5 M NaCl, 0.25 M HEPES, 75 mM Na_2_HPO_4_, pH 7.1). Cells were cultured with 5% CO_2_ at 37°C for 18 h, and changed with OptiMEM/10% FBS. Two days after co-transfection, cells and medium were collected, centrifuged at 1140 *g* for 15 min, and then resuspended in 150 mM NaCl/20 mM Tris pH 8.0 at 5 × 10^6^ cells/ml. The cell suspension was further treated with 0.54% deoxycholate (Sigma, St. Louis, MO, USA) and 50 U/ml Benxonase (Sigma) at 37°C for 1 h. Following centrifugation at 3000 *g* at room temperature for 20 min, supernatants were subjected to a cycle of freeze − thaw, and then centrifuged again at 10,000 *g* at 4°C for 30 min. The supernatant was collected, filtered through a 1-μm disk filter (Fisher, Pittsburgh, PA, USA), and then run by gravity through a heparin agarose type I column (Sigma) pre-equilibrated with phosphate buffer saline/1 mM MgCl_2_/2.5 mM KCl phosphate-buffered saline (PBS-MK). After four washes with 5 ml PBS-MK each, rAAV viruses were eluted by 9 ml of 1 M NaCl/PBS-MK. The first 2 ml was discarded. The next 7 ml was collected, desalted by running through a Centricon Plus-20/Biomax-100 (Fisher) with four changes of lactated Ringer’s solution, then concentrated by centrifugation at 3000 *g* at room temperature, and the elution was collected. Dot blot indicated that the titer of the packaged virus was in the range of 10^9^–10^10^ transducing units/ml.

### Animal surgery

Rats (*n* = 10/group) were anesthetized intramuscularly (IM) with 43 mg/kg of ketamine, 8.6 mg/kg of xylazine, and 1.5 mg/kg of acepromazine in physiological saline (0.9% NaCl) and placed in a Kopf rat stereotaxic apparatus (David Kopf Instruments, Tujunga, CA, USA) with the upper incisor bar at −3.8 mm below the interaural line. A Hamilton microsyringe (Hamilton, Reno, NV, USA) was then lowered unilaterally into the VTA at a 9° from the midsaggital plane in relation to bregma: [anteroposterior (AP) −5.3 mm, mediolateral (ML) + 1.3 mm, and dorsoventral (DV) −8.1 and 8.5 mm from skull (Paxinos and Watson, 1998; McMahon et al., 2001; Shank et al., 2007)]. The rAAV-5-HT_2A_R-Flag (2 μl, 10^9^–10^10^ transducing units/ml) or lactated ringer’s solution vehicle control (2 μl) was infused into the VTA (*n* = 10 per group) using the UMP II infusion pump (WPI, Sarasota, FL, USA) at a rate of 18 nl/min; the infusion lasted 2 h. Following infusion, the needle was left in place for 10 min followed by withdrawal from the brain and wound closure. Rats received a single injection (IM) of 300,000 U of sodium ampicillin after surgery and were allowed 1 week to recover, during which time they were handled and weighed daily.

### Behavioral procedures

#### Apparatus

Locomotor activity was quantified using a modified open-field activity system under low-light conditions (San Diego Instruments, San Diego, CA, USA). Each enclosure consisted of a clear Plexiglas open-field (40 cm × 40 cm × 40 cm) and a 4 × 4 photobeam matrix located 4 cm above the cage floor for the measurement of horizontal activity; each monitor was housed within sound-attenuating chambers. A second horizontal row of 16 photobeams located 16 cm from the floor allowed the measurement of rearing. Activity counts were made by the control software (Photobeam Activity Software, San Diego Instruments, San Diego, CA, USA) and stored for statistical evaluation. Video cameras located above the enclosures were used to monitor activity continuously without disruption of behavior.

#### Effects of 5-HT_2A_R overexpression on basal and cocaine-evoked locomotor activity

On Day 7 following surgery, animals were placed in activity monitors and horizontal activity and rearing were recorded for 60 min, followed by return to the animal colony. Additionally, activity was again measured in these same animals on Days 14 and 21 following surgery for 60 min on each day. On Day 21, following the measurement of basal locomotor activity, all animals were challenged with 15 mg/kg of cocaine [(-)-cocaine HCl salt; National Institute on Drug Abuse, Research Triangle, NC, USA dissolved in 0.9% NaCl], a dose that consistently produces hyperactivity in our laboratory (McCreary and Cunningham, 1999; De La Garza and Cunningham, 2000; Liu and Cunningham, 2006; Cunningham et al., 2013). Immediately following injection, horizontal activity and rearing were measured for 60 min.

Both horizontal activity and rearing counts were totaled for each animal in 10-min time bins and across the 60-min test sessions. All data are presented as mean horizontal activity counts or rearing counts (±SEM). For basal locomotor activity, a two-way ANOVA was used to analyze the effects of intra-VTA pretreatment (control or rAAV-5-HT_2A_R-Flag; factor 1) and day (Days 7, 14, 21; factor 2) with pretreatment as a between-subjects factor and day as a within-subjects factor. Planned comparisons for each test day were made with a Student’s *t*-test with a Bonferroni correction. To analyze the time course of cocaine-evoked activity on Day 21, a two-way ANOVA with factors of intra-VTA pretreatment (between-subjects) and time (within-subjects) was utilized followed by planned comparisons at each time point using a Student’s *t*-test with a Bonferroni correction. Differences in the mean total hyperactivity observed for the 60-min period following cocaine injection on Day 21 were analyzed with a Student’s *t*-test. All statistical tests were determined using SAS for Windows (Version 8.1) with an experiment wise α = 0.05.

#### Histology and transgene detection

At the end of behavioral testing on Day 21, animals were deeply anesthetized with an intraperitoneal (IP) injection of pentobarbital (Sigma, 100 mg/kg) and transcardially perfused with PBS followed by 3% buffered paraformaldehyde. Brains were then removed, blocked at the mid-pons, and post-fixed in paraformaldehyde at room temperature for 2 h. Tissue was then cryoprotected in 30% sucrose solution at 4°C for 48 h. Brains were frozen with crushed dry ice and stored at −80°C. Coronal sections (50 μm) were prepared with a Leica cryostat (CM 1850) at −20°C and processed to verify microinjection placement and transgene expression using immunohistochemistry (see below). Data obtained from rats with infusion sites outside of the VTA were excluded from analysis.

To validate the ability of the rAAV construct to establish expression of 5-HT_2A_R and Flag within the VTA, we employed immunohistochemical techniques using diaminobenzidine detection and light microscopy as described previously (Allen and MacPhail, 1991; Ross et al., 2006; Shank et al., 2007). Briefly, sections were blocked with a solution containing 1.5% normal goat serum (Vector Laboratories, Burlingame, CA, USA) in PBS with 0.4% Triton-X (PBS-T; Sigma), followed by incubation in PBS-T containing a polyclonal antibody for either the 5-HT_2A_R (1:1000; courtesy of Dr. Bryan Roth, Case Western University Cleveland, OH, USA; Garlow et al., 1993; Roth et al., 1995; Cornea-Hebert et al., 2002; Nocjar et al., 2002; Bubar et al., 2005; Ross et al., 2006) or Flag peptide (1:1000; Sigma). Sections were washed in PBS, incubated in PBS-T containing biotinylated goat-anti-rabbit IGg (1:400; Vector), incubated in an avidin-biotin-horseradish peroxidase complex (Vector), washed in TRIS buffer and developed in 3,3′-diaminobenzidine (0.5 mg/ml; Sigma) with 0.005% H_2_O_2_. Sections were mounted onto slides and coverslipped, followed by visualization with an Olympus Vanox-T AH2 microscope and image capture using a Pixera Professional camera (VCS10132; Sherwood Dallas, Co., Dallas, TX, USA) that was connected to a personal computer. Images of the VTA ipsilateral and contralateral to the injection site were captured and each image was subsequently cropped to a fixed-size rectangle of 420 × 1238 pixels located within the parabrachial-paranigral subnuclei of the VTA (Phillipson, 1979; Swanson, 1982) for comparative analyses. In accordance with recent image guidelines (Couzin, 2006), Adobe Photoshop (Adobe Systems, San Jose, CA, USA) was employed to mask dark shadows arising as injection artifacts on several sections.

The fixed-size images of the VTA were analyzed using a program written in Matlab (MathWorks, Inc., Natick, MA, USA) and the red channel of the image data was used for analysis (Hillman, 1984; Pollandt et al., 2005; Liu et al., 2007). The intensity histogram was computed, producing a bell-shaped curve, skewed toward the dense side by the presence of darker (stained) pixels, which occupy a small fraction of the image area. The non-stained tissue density was modeled by fitting a normal curve to the upper portion of the histogram, using the Marquard non-linear least-squares method. The fitted curve was subtracted from the observed histogram on the dense side of the peak, providing an estimate of the intensity distribution of staining. A threshold was selected that was 2.6 standard deviations below the mean of the fitted background curve. Pixels darker than the threshold were considered to be stained and were displayed as a map for visual confirmation. The number of such pixels was counted to quantify immunolabeling (Hillman, 1984; Pollandt et al., 2005; Liu et al., 2007). Total immunolabeling was determined as the sum of stained pixels weighted by their density below the staining threshold and was calculated from VTA images ipsilateral and contralateral to the infusion site. The difference in total immunolabeling from the ipsilateral minus contralateral VTA from each animal was compared between infusion groups with an unpaired Wilcoxon test.

To explore localization of 5-HT_2A_R within VTA cells and co-localization of 5-HT_2A_R in DA neurons, confocal microscopy (Bubar and Cunningham, 2007; Bubar et al., 2011; Anastasio et al., 2013) was utilized to study double-label immunofluorescence with previously validated antibodies for the 5-HT_2A_R (Garlow et al., 1993; Roth et al., 1995; Cornea-Hebert et al., 2002; Nocjar et al., 2002; Bubar et al., 2005) and tyrosine hydroxylase (TH; Browning et al., 2005). Methods for immunofluorescence were similar to those described above with a few minor modifications. A separate cohort of rats (*n* = 7) was unilaterally infused with lactated ringer’s solution control or rAAV-5-HT_2A_R-Flag (as described above) and sacrificed 4 weeks following infusion. Sections (25 μm) were prepared using the Leica cryostat, followed by several washes and incubation in blocking serum (PBS-T plus 1.5% goat serum) as described above. Single sections were then incubated in PBS-T containing both the 5-HT_2A_R antibody (1:1000) and a monoclonal antibody for TH (1:2500; Immunostar). Sections were then washed in PBS and incubated in PBS-T containing secondary fluorescent goat-anti-rabbit (1:2000; Alexa fluor 555; Invitrogen, Carlsbad, CA, USA) and goat-anti-mouse (1:2000; Alexa fluor 488; Invitrogen) antibodies at room temperature. Last, sections were mounted as described above and labeling visualized at the UTMB Infectious Disease Optical Imaging Core using a Zeiss LSM 510 Meta confocal microscope and image capture with LSM 5 imaging software (Carl Zeiss Microimaging, Thornwood, NY, USA) that was connected to a personal computer.

## Results

### Effects of 5-HT_2A_R overexpression on basal and cocaine-evoked activity

To test the hypothesis that overexpression of the 5-HT_2A_R in the VTA enhances basal or cocaine-evoked hyperactivity, male rats (*n* = 10/group) were pretreated with intra-VTA infusion of either lactated Ringer’s solution (control) or rAAV-5-HT_2A_R-Flag (virus). Of these, nine control rats exhibited needle placements positioned in the VTA (see below); one animal contained a needle placement outside of the VTA, and was thus excluded. Of virus-pretreated animals, five exhibited proper VTA placement as well as virally mediated overexpression of the 5-HT_2A_R (see below). One virus-pretreated animal exhibited overexpression in the hypothalamus as a result of incorrect placement, and four animals did not exhibit 5-HT_2A_R overexpression; these animals were excluded from analysis.

**Figure 1:**
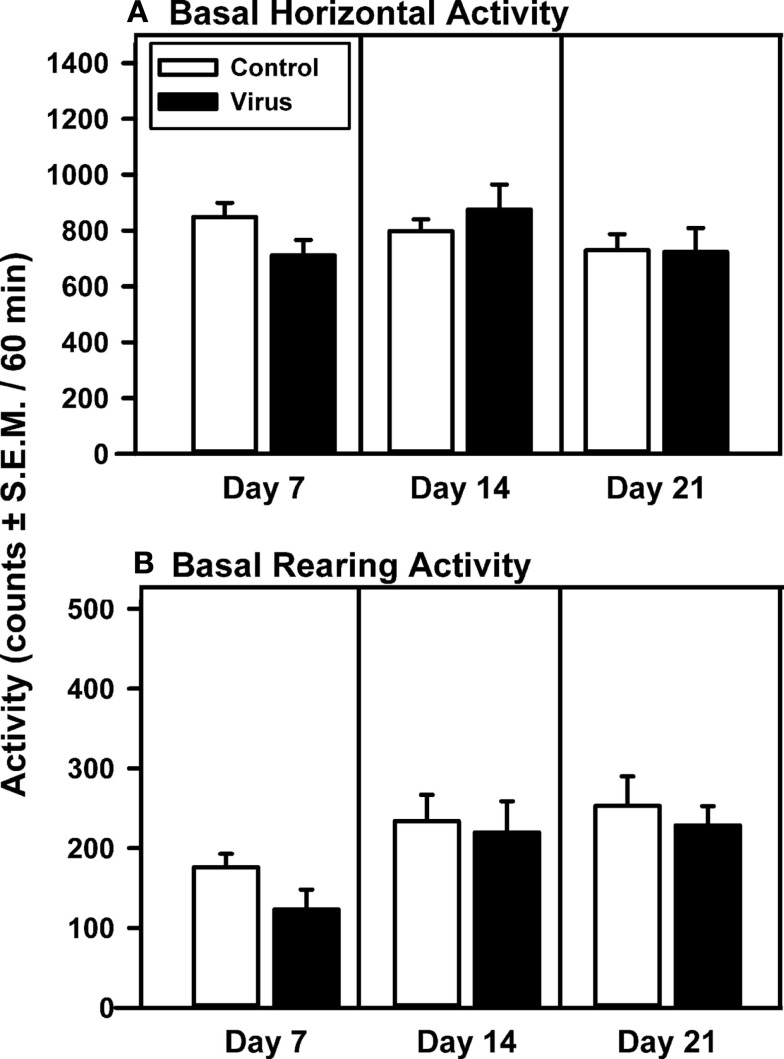
**Basal levels of motility are unchanged following intra-VTA infusion of rAAV-5-HT_2A_R-Flag**. Mean total horizontal **(A)** or rearing activity **(B)** [counts/60 min (±SEM)] were recorded 7, 14, and 21 days following intracerebral pretreatment with 2 μl of vehicle (Control; open bar; *n* = 9) or rAAV-5-HT_2A_R-Flag (Virus, filled bar; *n* = 5). Both pretreatment groups exhibited similar levels of horizontal and rearing activity on each day analyzed.

Basal locomotor activity measured on Days 7, 14, and 21 was analyzed for control animals with proper VTA placements and virus-pretreated animals with overexpression confined to the VTA (Figure 1). The levels of basal locomotor activity observed, regardless of pretreatment, were similar to levels of locomotor activity evoked upon saline injection in previous studies (McMahon and Cunningham, 2001; McMahon et al., 2001; Filip and Cunningham, 2002; Bubar et al., 2003). There was no main effect of pretreatment (*F*_1, 41_ = 0.11, *p* = 0.747), day (*F*_2, 41_ = 3.17, *p* = 0.06), or a pretreatment × day interaction (*F*_2, 41_ = 3.18, *p* = 0.06) observed for basal horizontal activity on Days 7, 14, and 21 after viral injections. *A priori* comparisons indicated that the basal horizontal activity did not differ between pretreatment groups on any test day (Figure 1A). For basal rearing activity, a main effect of day (*F*_2, 41_ = 10.97, *p* = 0.0004) in the absence of a main effect of pretreatment (*F*_1, 41_ = 0.65, *p* = 0.435) or a pretreatment × day interaction (*F*_2, 41_ = 0.46, *p* = 0.638) was observed (Figure 1B); *a priori* comparisons between treatment groups failed to indicate significant differences in basal rearing activity between control and virus treatment groups on any given test day. Levels of basal activity in animals with misplaced rAAV-5-HT_2A_R-Flag infusions outside of the VTA did not differ from control animals on days 7, 14, or 21 (data not shown; *p* > 0.05).

**Figure 2:**
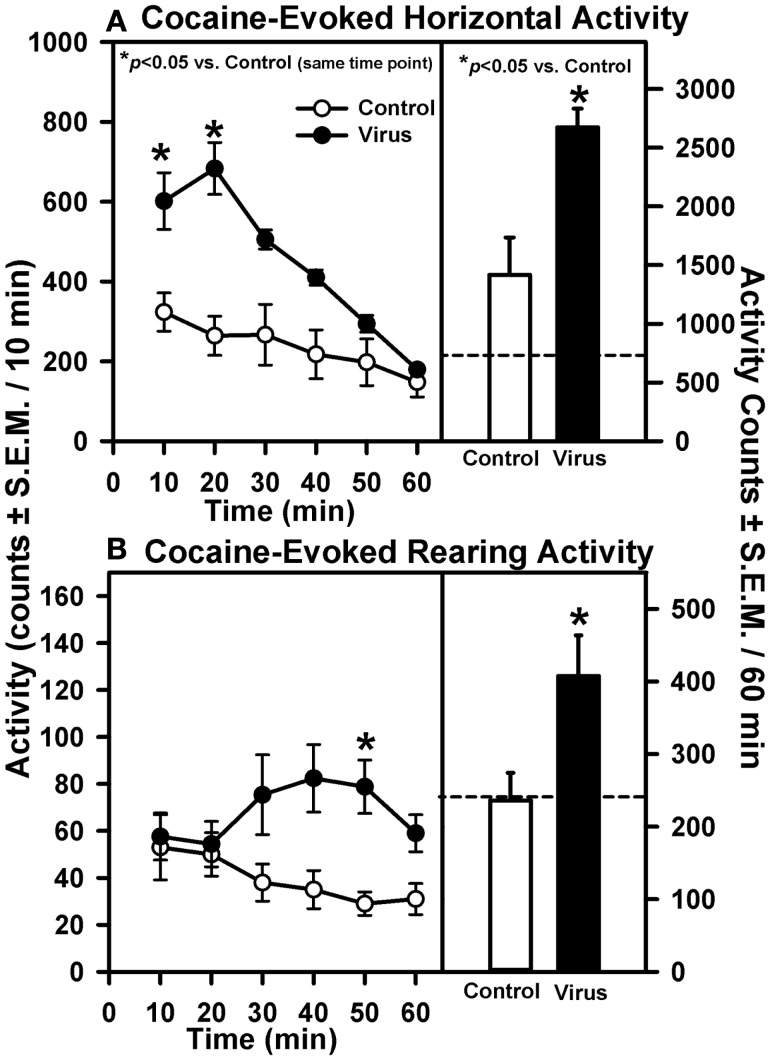
**Intra-VTA infusion of rAAV-5-HT_2A_R-Flag is associated with elevated cocaine-induced motility**. Data represent the mean total horizontal **(A)** or rearing activity **(B)** in rats induced upon injection with cocaine (15 mg/kg, ip) 21 days after a 2 μl infusion of vehicle (Control; open symbols; *n* = 9) or rAAV-5-HT_2A_R-Flag (Virus; filled symbols; *n* = 5). Left panels represent the *timecourse* across the 60 min session divided into 10 min bins [mean activity counts/10 min (±SEM)]; *p < 0.008 vs. control at same time point. Right panels illustrate mean total horizontal **(A)** or rearing activity **(B)** for the entire 60 min session [counts/60 min (±SEM); *p < 0.05 vs. control. Dashed line represents average level of basal horizontal or rearing activity determined in the 60 min period prior to cocaine injection (see Figure 1).

On Day 21 following the measurement of basal locomotor activity, all animals were challenged with 15 mg/kg of cocaine and activity was recorded for 60 min (Figure 2). A main effect of pretreatment (*F*_1, 83_ = 7.84, *p* = 0.016), time (*F*_5, 83_ = 28.37, *p* < 0.0001), and a pretreatment × time interaction (*F*_5, 83_ = 28.37, *p* < 0.0001) were observed for cocaine-evoked horizontal activity measured in 10-min time bins during the 60-min test (Figure 2A, left panel). *A priori* comparisons indicated that viral pretreatment was associated with significantly greater cocaine-evoked horizontal activity during each of the first two time bins (10 and 20 min) of the test period as compared to control animals (*p* < 0.008/comparison). A trend for increased cocaine-evoked horizontal activity was observed but not statistically significant at the 30 min (*p* = 0.04) and 40 min time bins (*p* = 0.04). The *a priori* analysis indicated that virus pretreatment was associated with significantly greater levels of cocaine-evoked horizontal activity totaled for the entire 60-min test session (Figure 2A, right panel; *p* < 0.05). Levels of cocaine-evoked horizontal activity in animals with misplaced rAAV-5-HT_2A_R-Flag infusions outside of the VTA did not differ from control animals (data not shown; *p* > 0.05).

Rearing activity was also measured following cocaine injection on Day 21. A main effect of pretreatment (*F*_1, 83_ = 6.61, *p* = 0.025) and a pretreatment × time interaction (*F*_5, 83_ = 3.13, *p* = 0.014), but not a main effect of time (*F*_5, 83_ = 0.82, *p* = 0.538), were observed for cocaine-evoked rearing activity measured in 10-min time bins during the 60-min test (Figure 2B). *A priori* planned comparisons indicated that the viral pretreatment was associated with greater cocaine-evoked rearing activity at the 50 min time bin (*p* < 0.008/comparison), with the comparisons made at the 30 min (*p* = 0.047), 40 min (*p* = 0.01), and 60 min time bins (*p* = 0.023) of the test period approaching statistical significance (Figure 2B, left panel). The *a priori* analysis indicated that virus pretreatment was associated with significantly greater levels of cocaine-evoked rearing activity totaled across the entire 60-min test session compared to control animals (Figure 2B, left panel; *p* < 0.05). Levels of cocaine-evoked rearing activity in animals with misplaced rAAV-5-HT_2A_R-Flag infusions outside of the VTA did not differ from control animals (data not shown; *p* > 0.05).

### 5-HT_2A_R and Flag immunohistochemistry

**Figure 3:**
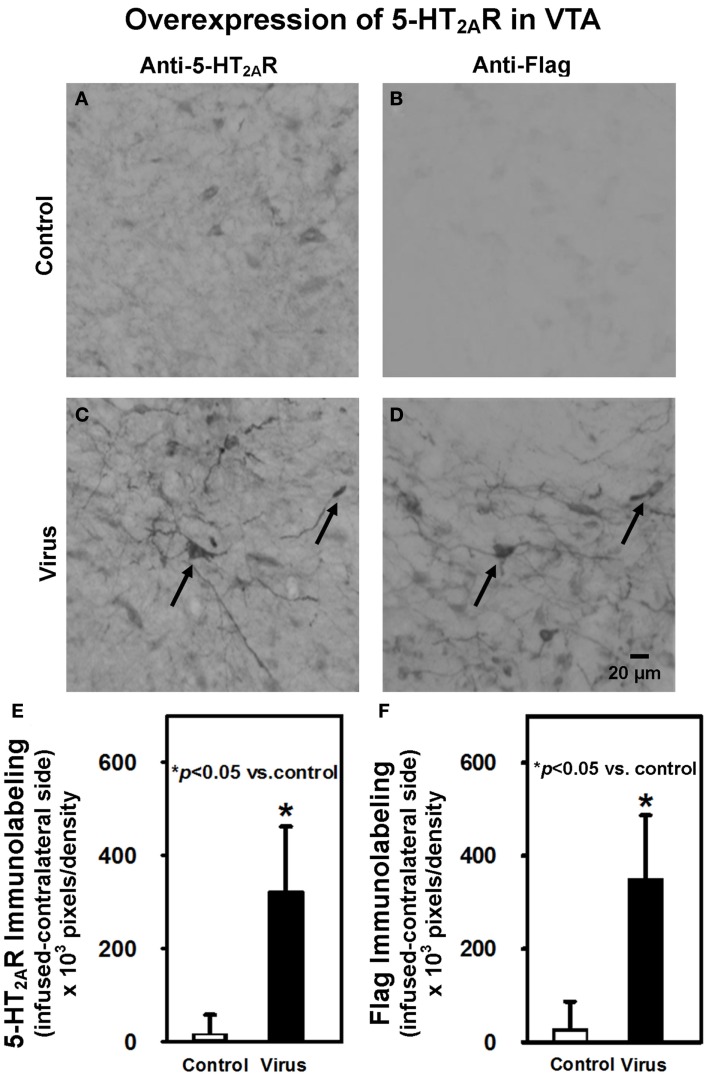
**Intra-VTA infusion of rAAV-5-HT_2A_R-Flag is associated with overexpression of 5-HT_2A_R and Flag peptide immunolabeling**. Shown are representative photomicrographs of **(A)** anti-5-HT_2A_R antibody labeling of VTA neurons in control animals; **(B)** anti-Flag antibody labeling of VTA neurons in control animals; **(C)** anti-5-HT_2A_R antibody labeling of VTA neurons in virus animals infused with rAAV-5-HT_2A_R-Flag; **(D)** labeling with anti-Flag antibody in an immediately adjacent tissue section of virus-pretreated animal indicates co-localization of 5-HT_2A_R and Flag. **(E)** Quantification of net 5-HT_2A_R immunoreactivity (±SEM) in VTA of control animals (open bar; *n* = 9) vs. virus animals infused with rAAV-5-HT_2A_R-Flag-(filled bar; *n* = 5). Animals that previously received intra-VTA viral infusion exhibited greater 5-HT_2A_R immunoreactivity than control animals, confirming viral overexpression of 5-HT_2A_R. **(F)** Quantification of net Flag immunoreactivity (±SEM) in VTA of control animals (open bar; *n* = 9) vs. virus animals infused with rAAV-5-HT_2A_R-Flag (filled bar; *n* = 5). Animals that previously received intra-VTA viral infusion exhibited greater Flag immunoreactivity than those control animals, further confirming viral overexpression of 5-HT_2A_R. Scale bar = 20 μm. *p < 0.05 vs. control.

Following the completion of behavioral testing on Day 21, animals were sacrificed and immunohistochemistry performed to confirm overexpression of 5-HT_2A_R and expression of Flag in the VTA (Figure 3). A representative photomicrograph depicting 5-HT_2A_R immunolabeling in the VTA ipsilateral to the infusion site from a control animal illustrates that the majority of the 5-HT_2A_R immunoreactivity seems to be confined to cell bodies, with little fiber labeling (Figure 3A). Control animals exhibited little Flag background labeling (Figure 3B). In contrast, 5-HT_2A_R immunolabeling in the ipsilateral VTA from a virus animal infused with rAAV-5-HT_2A_R-Flag (Figure 3C) illustrates a distinct pattern of 5-HT_2A_R immunolabeling characterized by robust 5-HT_2A_R immunoreactivity in both cell bodies and fibers. A brain section labeled with the anti-Flag antibody and adjacent to that shown in Figure 3C shows a similar expression of immunoreactivity, with labeled cell bodies as well as fibers (Figure 3D). Additionally, the arrows indicate labeled cells in Figures 3C,D seem to be identical, providing further evidence of successful overexpression.

A comparison of the 5-HT_2A_R immunoreactivity quantified in the VTA (Figure 3E) was made using an unpaired Wilcoxon test. Control animals exhibited similar, moderate levels of 5-HT_2A_R immunoreactivity in the VTA ipsilateral (Figure 3A) and contralateral (data not shown) to the infusion site. Infusion of rAAV-5-HT_2A_R-Flag resulted in overexpression of 5-HT_2A_R in the ipsilateral (Figure 3C), but not contralateral, VTA (data not shown). The total net immunolabeling (ipsilateral immunolabeling – contralateral immunolabeling) was then calculated in individual animals in order to normalize 5-HT_2A_R overexpression to basal 5-HT_2A_R levels in the brain hemisphere contralateral to viral infusion. This total net immunolabeling was then compared between pretreatment groups and the results indicate that greater levels of 5-HT_2A_R expression were exhibited in virus-pretreated animals (*p* < 0.05; Figure 3E). Quantification of Flag immunoreactivity with this same procedure also revealed robust levels of Flag labeling in virus-pretreated animals, as compared to control animals (*p* < 0.05; Figure 3F), further confirming successful expression of the transgene. Levels of 5-HT_2A_R and Flag immunoreactivity in animals with misplaced rAAV-5-HT_2A_R-Flag infusions outside of the VTA did not differ from control animals (data not shown; *p* > 0.05).

**Figure 4:**
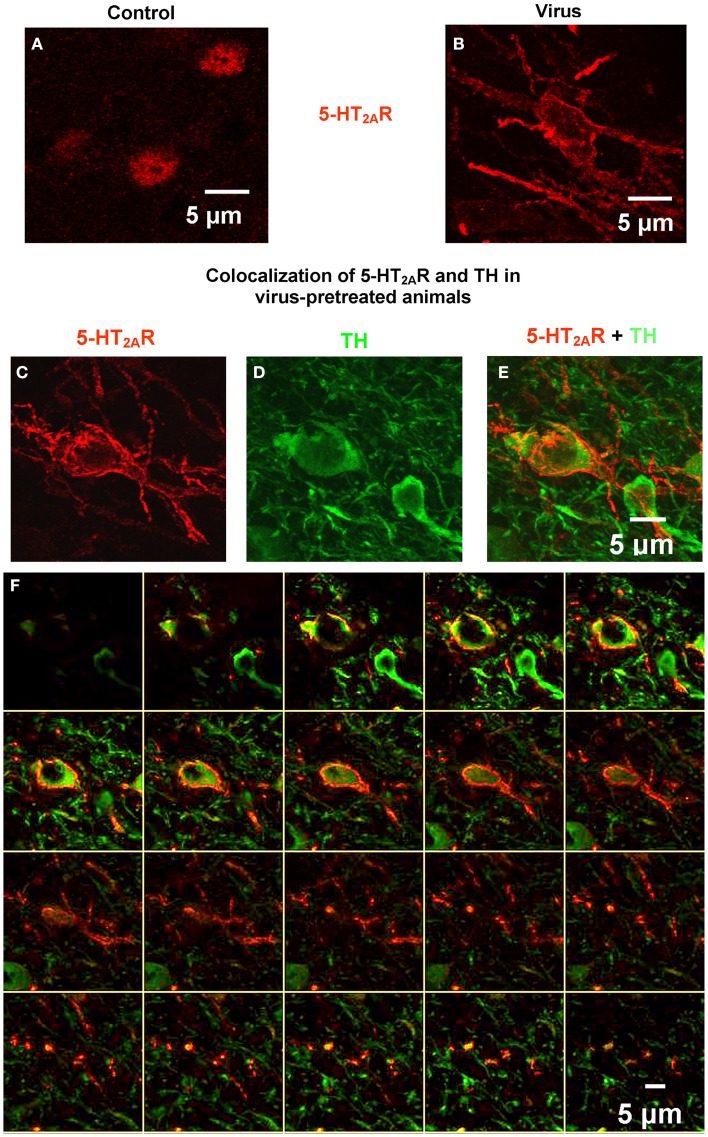
**Confocal microscopy demonstrates robust 5-HT_2A_R labeling in the VTA and co-localization in cells immunopositive for tyrosine hydroxylase (TH)**. **(A)** Representative composite confocal image (24 slices, 0.68 μm/slice) demonstrates 5-HT_2A_R immunoreactivity (red) in VTA neurons from control animals. **(B)** Representative composite confocal image (24 slices, 0.7 μm/slice) demonstrates 5-HT_2A_R immunoreactivity (red) in VTA neurons from virus animals pretreated with rAAV-5-HT_2A_R-Flag. **(C–F)** Representative composite confocal image (24 slices, 0.71 μm/slice) demonstrating co-localization of 5-HT_2A_R and TH in VTA neurons from virus animals pretreated with rAAV-5-HT_2A_R-Flag. In the same tissue section, there is robust 5-HT_2A_R immunoreactivity **(C)** and TH immunoreactivity **(D)** that is colocalized to the same cells **(E)**. **(F)** Contains 20 of the *Z*-sections that make up the composite image. Scale bar = 5 μm.

Confocal microscopy was utilized to analyze tissue sections processed for double-labeled 5-HT_2A_R and TH immunofluorescence in the VTA in order to assess localization of 5-HT_2A_R to DA neurons (Figure 4) from animals pretreated with vehicle (Figure 4A) or AAV-5-HT_2A_R-Flag (Figures 4B–F). Figure 4A demonstrates a composite confocal image (24 sections, 0.68 μm/slice) of 5-HT_2A_R immunoreactivity from the VTA of a control animal. Immunolabeling is predominantly confined to cell body regions (Figure 4A) in keeping with our observation of 5-HT_2A_R staining using DAB (above; Figure 3) and previous studies using the same anti-5-HT_2A_R antibody (Nocjar et al., 2002; Bubar et al., 2005). Figure 4B demonstrates a composite confocal image (24 sections, 0.70 μm/slice) of 5-HT_2A_R immunoreactivity in the VTA of an animal infused with rAAV-5-HT_2A_R-Flag. Overexpression is indicated by the robust immunoreactivity in both cell bodies and fibers (Figure 4B). Figures 4C–F represents composite confocal images of double-label immunofluorescence from an animal infused with rAAV-5-HT_2A_R-Flag. Figure 4C demonstrates a composite confocal image (24 sections, 0.71 μm/slice) of 5-HT_2A_R immunoreactivity in the VTA of an animal infused with AAV-5-HT_2A_R-Flag, and Figure 4D demonstrates immunoreactivity for TH in the same tissue section as that shown in Figure 4C. The composite image represents localization of 5-HT_2A_R immunoreactivity in a TH-positive cell (Figure 4E). Additionally, 20 of the serial *Z*-sections that comprise the composite image in Figure 4E are shown in Figure 4F (Bubar and Cunningham, 2007; Bubar et al., 2011; Anastasio et al., 2013).

## Discussion

The present study is the first to demonstrate that overexpression of 5-HT_2A_R protein in the VTA enhances the behavioral effects of cocaine following successful virally mediated overexpression of the 5-HT_2A_R in the adult rat. Intra-VTA transduction with the rAAV-5-HT_2A_R-Flag vector, which produced quantifiable overexpression of 5-HT_2A_R and appearance of the Flag protein in VTA neurons, had little effect on basal levels of motor activity, but significantly enhanced cocaine-evoked motility relative to controls. These results are in line with an overall facilitatory role for the 5-HT_2A_R in mediating cocaine-evoked behaviors (see, Bubar and Cunningham, 2008; Nic Dhonnchadha and Cunningham, 2008) and support the hypothesis that the VTA is a key site of action for the 5-HT_2A_R to control the behavioral effects of cocaine.

The current results revealing that overexpression of 5-HT_2A_R in the VTA enhances cocaine-evoked hyperactivity are in accordance with a previous study from our laboratory demonstrating that intra-VTA microinjection of the selective 5-HT_2A_R antagonist M100907 blocked cocaine-evoked hyperactivity (McMahon et al., 2001). These effects parallel those of systemic injection of 5-HT_2A_R ligands, as 5-HT_2A_R *antagonists* block and 5-HT_2A_R *agonists* enhance the hypermotive effects of cocaine (McMahon and Cunningham, 2001; Fletcher et al., 2002; Filip et al., 2004) and other stimulants (Auclair et al., 2004; Herin et al., 2005). These data implicate the VTA as a critical site of action for the positive modulatory control of 5-HT_2A_R over psychostimulant-evoked motor activity. This stimulatory role for VTA 5-HT_2A_R upon cocaine-evoked hypermotility appears to occur in the absence of an apparent tonic regulatory influence of this receptor on motor activity. Overexpression of the 5-HT_2A_R in the VTA has no effect upon basal levels of motility evoked upon exposure to the activity monitors. These data are consistent with studies in the literature demonstrating that selective blockade of 5-HT_2A_R in the VTA does not alter spontaneous locomotor behavior (McMahon et al., 2001; Auclair et al., 2004). However, we have observed the enhancement of motor activity upon intra-VTA infusion of the non-selective 5-HT_2A_R agonist DOI (Herin et al., unpublished observations). Together, these data indicate that, despite robust enhancement of 5-HT_2A_R immunoreactivity in both cell bodies and fibers relative to controls, elevated expression of the 5-HT_2A_R in the VTA alone is not sufficient to induce overt alterations in basal motor activation. However, the elevated VTA 5-HT_2A_R expression generates an augmented and positive modulatory effect over cocaine-evoked hyperactivity.

Several characteristics of the 5-HT_2A_R may account for the low levels of basal 5-HT_2A_R function. For example, the 5-HT_2A_R exhibits moderate affinity for 5-HT (Peroutka, 1986; Rothman et al., 2000; Leysen, 2004) and possesses modest constitutive activity in the absence of ligand binding (Berg et al., 2005). In addition, although the 5-HT_2A_R is thought to primarily localize to somata or dendrites postsynaptic to 5-HT terminals, ultrastructural localization studies indicate that the receptor prominently localizes to the cytoplasm, rather than the plasma membrane, and is primarily found in extrasynaptic regions (Cornea-Hebert et al., 1999; Doherty and Pickel, 2000). Such localization patterns suggest that a component of 5-HT actions at the 5-HT_2A_R may occur via paracrine or volume transmission which may be minimal at baseline (Miner et al., 2000; Jansson et al., 2001). Thus, activation of the 5-HT_2A_R receptors in VTA may only occur during periods of stimulated 5-HT release like that evoked following systemic cocaine administration (Chen and Reith, 1994).

The differential effects of 5-HT_2A_R antagonists delivered into the VTA upon basal vs. cocaine-stimulated motor activity have been attributed to their efficacy to alter the activation status of the DA mesoaccumbens pathway to control locomotor activity (Kelly and Iversen, 1976; Broderick et al., 2004), and are in accordance with a prevailing hypothesis that the 5-HT_2A_R modulates DA mesocorticoaccumbens neurotransmission only under “stimulated” conditions (Schmidt et al., 1992; De Deurwaerdere and Spampinato, 1999; Di Giovanni et al., 1999; Bonaccorso et al., 2002; Kuroki et al., 2003; Auclair et al., 2004). In accordance with the lack of effects of intra-VTA administration of 5-HT_2A_R antagonists on basal levels of motor activation (McMahon et al., 2001; Auclair et al., 2004), local infusion of 5-HT_2A_R antagonists into the VTA failed to alter basal DA release in the NAc (Auclair et al., 2004), nor did perfusion of 5-HT_2A_R antagonists alter firing rates of VTA DA neurons in a midbrain slice preparation (Olijslagers et al., 2004). Conversely, intra-VTA 5-HT_2A_R antagonist administration significantly blocked systemic cocaine- (McMahon et al., 2001) and amphetamine-evoked hypermotility (Auclair et al., 2004) and associated amphetamine-evoked NAc DA release (Auclair et al., 2004). Thus the selective effects of VTA 5-HT_2A_R overexpression upon cocaine-evoked as opposed to basal motor activity are likely due to 5-HT_2A_R-mediated facilitation of DA mesoaccumbens neurotransmission under stimulated vs. tonic conditions, respectively.

The 5-HT_2A_R is natively resident within DA and non-DAergic (GABA- or possibly glutamate-containing) neurons in the VTA (Doherty and Pickel, 2000; Ikemoto et al., 2000; Nocjar et al., 2002; Yamaguchi et al., 2007) although DA neurons comprise the majority of VTA neuronal cells (Swanson, 1982; Johnson and North, 1992; Ikemoto, 2007). Indeed, our confocal immunofluorescence studies (see Figure 4) provide evidence of 5-HT_2A_R overexpression in VTA DA neurons. Activation of elevated levels of 5-HT_2A_R resident in DA neurons consequent to cocaine-evoked elevations in 5-HT efflux (Chen and Reith, 1994) would be expected to increase activity of DA neurons (Pessia et al., 1994) and release of DA in terminal regions (De Deurwaerdere and Spampinato, 1999). As noted above, enhanced DA release in the NAc correlates positively with generation of hypermotility (Kelly and Iversen, 1976). Thus, overexpression of 5-HT_2A_R within DA neurons that project to the NAc would serve to enhance cocaine-evoked hyperactivity, as was observed in the present study. Although the overexpression of 5-HT_2A_R in the DA neurons aligns with the observed behavioral profile, 5-HT_2A_R overexpression also likely occurred in non-DAergic VTA neurons, presumably GABA interneurons and/or projection neurons, or possibly glutamate neurons (Yamaguchi et al., 2007; see Figure 4), since the constitutively active promoter utilized evokes gene expression in all neuronal cell types (Kaplitt et al., 2007; St Martin et al., 2007). Future studies employing a promotor that would direct viral expression to either DA, GABA, or glutamate neurons would help to discern the contribution of 5-HT_2A_R overexpression within the particular neuronal cell type to basal vs. cocaine-evoked locomotor activity.

The behavioral phenotype observed following rAAV-5-HT_2A_R-Flag infusion is most likely mediated by neurons intrinsic to the VTA. Measurable 5-HT_2A_R overexpression was confined to the site of infusion, and adjacent brain regions (especially, substantia nigra; data not shown) did not demonstrate patterns of 5-HT_2A_R overexpression. Second, in keeping with this observation, cells projecting to the VTA were not likely to be transduced, as the virus utilized in these studies (AAV-2) is not readily transported retrogradely following infusion into the brain (Chamberlin et al., 1998). Third, the cells transduced in the VTA fit the morphological profile suggestive of neurons, consistent with previous observations that AAV-2 does not readily transduce glial cells (Chamberlin et al., 1998). Fourth, while theoretically possible that viral vector transduction *per se* could evoke behaviorally relevant cellular changes, this possibility is highly unlikely. Previous studies have shown that transduction of neural tissue with AAV vectors does not alter the electrophysiological properties of neurons (Ehrengruber et al., 2001) or result in neurotoxicity (Lo et al., 1999), while animals infused intracranially with either vehicle or control viral vectors (Carlezon Jr. et al., 1997; Pliakas et al., 2001), including AAV (Landgraf et al., 2003), exhibit equally normal patterns of behavior. Basal locomotor activity did not differ between the animals infused with control vs. AAV in the present study, and the levels of activity observed were similar to that reported in previous studies following saline injection studies (McMahon and Cunningham, 2001; McMahon et al., 2001; Filip and Cunningham, 2002; Bubar et al., 2003). Finally, animals with rAAV-5-HT_2A_R-Flag infusions located outside the VTA exhibited levels of cocaine-evoked hyperactivity similar to that of control animals. Altogether, these data point to the overexpression of 5-HT_2A_R in the VTA as the most likely contributor to the observed enhancement of cocaine-evoked hyperactivity following rAAV-5-HT_2A_R-Flag infusion.

The results of present study suggest that expression levels of the 5-HT_2A_R in the VTA regulate vulnerability to the hypermotive effects of cocaine and support a possible role for VTA 5-HT_2A_R in modulating other behavioral effects of cocaine mediated by DA mesocorticoaccumbens circuitry. In addition to altering cocaine-evoked hyperactivity, systemic administration of 5-HT_2A_R antagonists has been shown to reduce the discriminative stimulus effects of cocaine (McMahon and Cunningham, 2001; Filip et al., 2006) and cocaine-evoked behavioral disinhibition (i.e., impulsivity; Anastasio et al., 2011; Fletcher et al., 2011), as well as to block expression of cocaine sensitization (Filip et al., 2004; Zayara et al., 2011). Furthermore, although the 5-HT_2A_R does not appear to modulate cocaine intake in the self-administration assay (Fletcher et al., 2002; Nic Dhonnchadha et al., 2009), 5-HT_2A_R antagonists have been shown to attenuate both cocaine- and cue-evoked reinstatement of cocaine-seeking (Fletcher et al., 2002; Nic Dhonnchadha et al., 2009). However, no studies have evaluated specifically the role of VTA 5-HT_2A_R receptors in cocaine-evoked behaviors other than locomotor hyperactivity (McMahon et al., 2001), though 5-HT_2A_R in the NAc (Zayara et al., 2011) and PFC (Pockros et al., 2011) have been implicated in sensitization and cue-evoked reinstatement of cocaine-seeking, respectively. Here we employed a single, low dose of cocaine (15 mg/kg) that consistently induces hypermotility in the absence of overt stereotypic behaviors (Herges and Taylor, 1998) to further establish a critical role for VTA 5-HT_2A_R in the hypermotive effects of cocaine. Even with the small sample size employed in the current study, the behavioral response to the single dose of cocaine produced a robust behavioral response with little variability. Our results combined with the knowledge regarding 5-HT_2A_R regulation of DA mesocorticoaccumbens activation provide the impetus to conduct more thorough investigations into the role of 5-HT_2A_R regulation in the VTA not only in the hypermotive effects of cocaine, but also more complex cocaine-associated behaviors. Furthermore, the methods established here utilizing rAAV-5-HT_2A_R-Flag to overexpress the 5-HT_2A_R can be employed to evaluate the role of elevated 5-HT_2A_R expression throughout the brain.

## Conflict of Interest Statement

The authors declare that the research was conducted in the absence of any commercial or financial relationships that could be construed as a potential conflict of interest.
